# Robust segregation of donor and recipient cells from single-cell RNA-sequencing of transplant samples

**DOI:** 10.3389/frtra.2023.1161146

**Published:** 2023-06-12

**Authors:** Gavin W. Wilson, Allen Duong, Sajad Moshkelgosha, Gary Bader, Shaf Keshavjee, Tereza Martinu, Stephen C. Juvet, Jonathan C. Yeung

**Affiliations:** ^1^ Latner Thoracic Surgery Research Laboratories, University Health Network, Toronto, ON, Canada; ^2^ The Donnelly Centre, University of Toronto, Toronto, ON, Canada; ^3^ Toronto Lung Transplant Program, Toronto General Hospital, Toronto, ON, Canada

**Keywords:** single-cell RNA-sequencing, solid-organ transplantation, bioinformatics, application, donor and recipient genotypes

## Abstract

**Background:**

Single-cell RNA-sequencing (scRNA-seq) technology has revealed novel cell populations in organs, uncovered regulatory relationships between genes, and allowed for tracking of cell lineage trajectory during development. It demonstrates promise as a method to better understand transplant biology; however, fundamental bioinformatic tools for its use in the context of transplantation have not been developed. One major need has been a robust method to identify cells as being either donor or recipient genotype origin, and ideally without the need to separately sequence the donor and recipient.

**Methods:**

We implemented a novel two-stage genotype discovery method (scTx) optimized for transplant samples by being robust to disparities in cell number and cell type. Using both *in silico* and real-world scRNA-seq transplant data, we benchmarked our method against existing demultiplexing methods to profile their limitations in terms of sequencing depth, donor and recipient cell imbalance, and single nucleotide variant input selection.

**Results:**

Using *in silico* data, scTx could more accurately separate donor from recipient cells and at much lower genotype ratios than existing methods. This was further validated using solid-organ scRNA-seq data where scTx could more reliably identify when a second genotype was present and at lower numbers of cells from a second genotype.

**Conclusion:**

scTx introduces the capability to accurately segregate donor and recipient gene expression at the single-cell level from scRNA-seq data without the need to separately genotype the donor and recipient. This will facilitate the use of scRNA-seq in the context of transplantation.

## Introduction

Droplet-based single-cell RNA-sequencing (scRNA-seq) enables gene expression profiling of thousands of individual cells and has led to novel insights into cancer and developmental biology. This technology forms the basis for the generation of detailed single-cell atlases of human organs at a previously unattainable resolution (1–4). Similarly, this technology has the promise of allowing for the study of solid-organ transplantation at this previously unattainable resolution (5). For example, interrogating the role of passenger donor immune cells within the recipient following kidney transplant (6, 7). However, in addition to identifying cell type, use of scRNA-seq for transplantation requires the additional step of identifying cells of donor genotype from cells of recipient genotype.

Recently, transplant scRNA-seq publications have used X- and Y-linked gene expression from sex-mismatched transplant samples to separate donor from recipient (8, 9), but this prevents study of non-sex-mismatched samples. Another previous study applied single nucleotide variant (SNV) deconvolution using demuxlet to profile kidney transplant immune cell persistence, but the major limitation is that demuxlet relies on matched genotyping data for the donor and recipient (7, 10, 11). Generation of matched genotyping data requires the collection and sequencing of isolated donor and recipient samples, greatly increasing the complexity and cost of an experiment, and separate donor and recipient tissues may not always be available. Another method might be to use previously published SNV-based genotype demultiplexing methods that were designed to identify pooled populations of cells from differing genetic backgrounds in order to differentiate donor and recipient cells (12–14). These SNV-based demultiplexing methods take advantage of expressed SNVs that can be identified in individual cells; however, these methods assume that the samples are pooled in equal proportions and that their cell types are similar. The latter is a critical assumption, as similar cell types will express similar gene expression programs, implying that they will express similar SNV loci that simplify the demultiplexing process. As transplant samples will vary in cell type and number, these methods may fail.

Indeed, the first major challenge with donor and recipient cell identification using SNVs from transplant scRNA-seq samples is that donor and recipient cell proportions and cell types may vary substantially; for example, donor cells are expected to be dominated by parenchymal cells and some passenger cells of hematopoietic origin, while recipient cells are expected to be mostly hematopoietic. The second major challenge with SNV-based demultiplexing methods is with the inherent technical challenges of scRNA-seq: (a) the sparsity and high false positive rate of SNV detection in a single cell, (b) the presence of cell–cell doublets (two cells in a single droplet), and (c) ambient RNA contamination (free-floating RNA molecules encapsulated into a droplet with a cell). Since the donor and recipient cell proportions can vary, SNVs that are specific to the less abundant genotype may be rare. We have previously demonstrated that germline SNVs can be called from scRNA-seq data with very low coverage; however, the false positive SNV rate is also increased (15). Therefore, SNV-based demultiplexing methods need to be robust to false positive SNVs. Cell–cell doublets and ambient RNA contamination both lead to scenarios where a cell with a particular genotype may be contaminated with SNVs derived from the other genotype. Depending on the combination of cells in the doublet and the amount of ambient RNA contamination, a singlet cell may be incorrectly identified as doublet or vice versa. The previously published demultiplexing methods have attempted to mitigate these issues but, again, they have not been tested in the transplant context.

In this article, we introduce scTx, a custom demultiplexing method that is specifically designed for transplant samples. We systematically benchmark scTx against two current popular genotype demultiplexing methods, Vireo and Souporcell, using simulated and biological transplant samples (12, 13). During benchmarking, we observed that Vireo and Souporcell performed inconsistently when there were large differences in the donor and recipient cell proportions, demonstrating that scTx is more robust than Vireo and Souporcell to the donor and recipient cell proportion imbalance and ambient RNA contamination. Finally, we demonstrate the accuracy of scTx on a variety of solid-organ transplant samples, including previously published kidney samples and novel lung transplant samples from bronchoalveolar lavages (BALs) and explanted lung allograft tissue.

## Materials and methods

### scRNA-seq alignment, cell identification, and SNV calling

For scRNA-seq alignment and SNV calling in scTx, we used our previously published tool, scSNV, which we demonstrated to have a reduced false positive call rate when calling rare germline SNVs (15). We processed all the samples using the default parameters and the human Ensembl build 102 for gene annotations and reference sequences (16).

### Demultiplexing scRNA-seq samples using scTx

**Figure 1:**
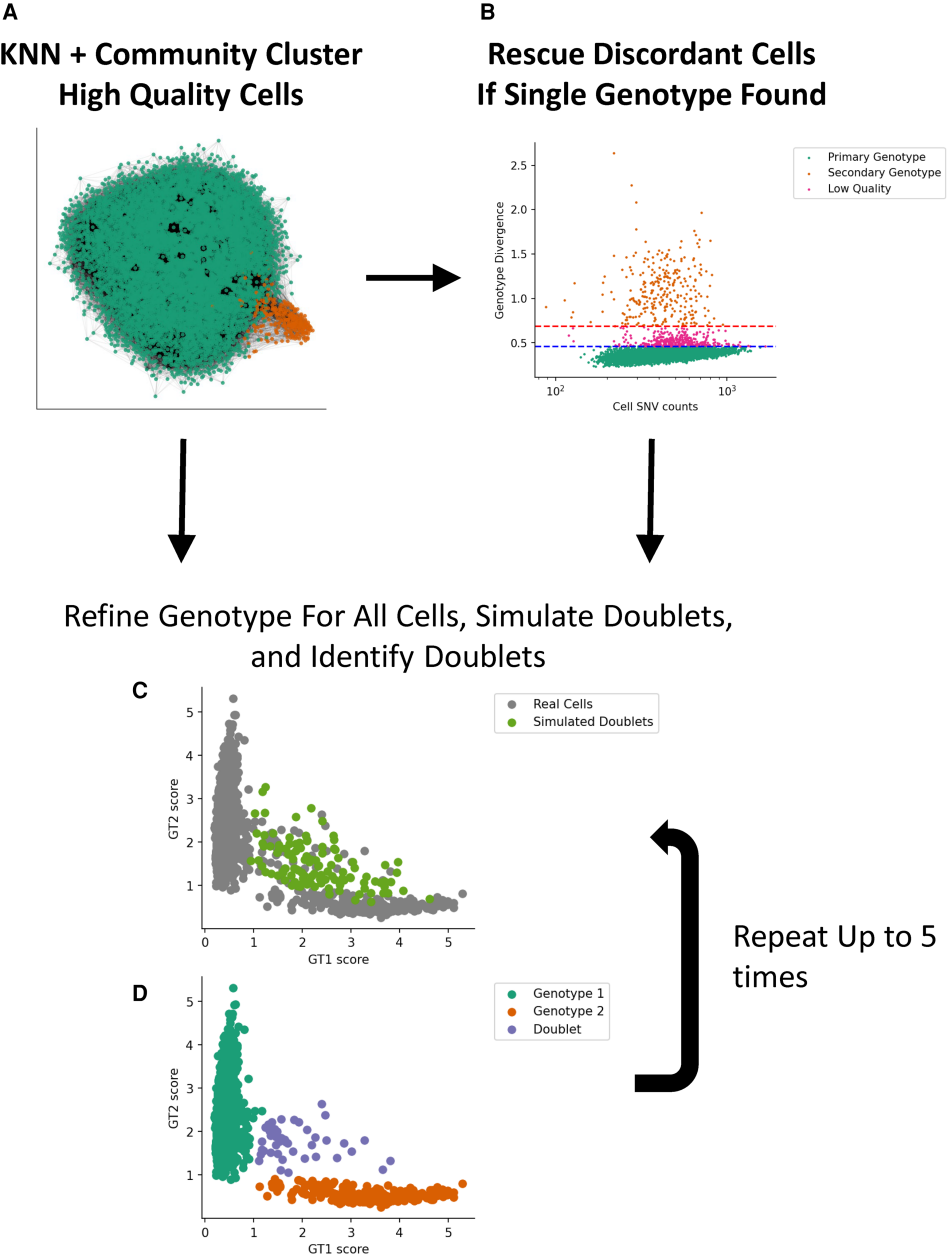
Implementation of a two-stage genotype discovery method. (**A**) Community clustering of a KNN graph based on allele fraction distances (see Materials and methods section). Only high-quality single cells that express a reasonable number of SNV loci using minor allele fractions are used in this step. (**B**) If a second genotype is not found, cells that differ substantially from the dominant GT using a genotype score metric are identified based on the median absolute deviation. If a second genotype is identified, we apply an algorithm that iteratively simulates and identifies cell–cell doublets (**C**) and refines the genotypes and cell assignments (**D**). KNN, k-nearest neighbor; GT, genotype.

We implemented a two-stage genotype discovery method. In the first stage, we cluster high-quality single cells that express a reasonable number of SNV loci using minor allele fractions (mAFs) with community clustering (Figure 1). If a second genotype is not found using our clustering approach, we assume the second genotype is at a low proportion and attempt to find it by looking for cells that differ substantially from the dominant genotype using a genotype score metric and apply thresholds based on the median absolute deviation. If a second genotype is still not found, our method stops processing the sample and indicates that a second genotype could not be found. After a second genotype is identified, we apply an algorithm that iteratively refines the genotypes, cell assignments, and simulates and identifies cell–cell doublets.

These steps can be performed interactively using an example available in the scTx code repository (https://github.com/GWW/sctx) and is detailed in Supplementary Material.

### Simulating lung transplant samples with doublet and ambient RNA contamination

Lung cells from the “Lung_367_T0” sample by Madissoon et al. and peripheral blood mononuclear cells (PBMC) cells from the PBMC 4k dataset from 10× Genomics were mapped with scSNV (17). Using these cells, we randomly generated three replicates of 12 lung and PBMC singlet cell mixtures each with a total of 4,000 cells. We used 25, 50, 75, 100, 150, 200, 300, 400, 500, 750, 1,000, and 2,000 total PBMC cells and the remaining 4,000 cells were from the lung cells. In addition to the 4,000 singlets, we generated lung/PBMC doublets by combining the reads of two cells at a rate of 10%. A function of scTx facilitates this by generating SNV calls from cell mixtures using the original files. To simulate ambient RNA contamination, scTx utilizes tag-collapsing introduced in scSNV and randomly gives each collapsed molecule a probability of being swapped to another randomly selected barcode. For each of the above replicates and mixing experiments, we generated an ambient RNA contaminated sample with rates of 0%, 10%, 20%, 30%, and 40%. This led to 3 × 12 × 5 = 180 simulated transplant experiments. The ambient RNA simulation we implemented in scTx properly swaps molecules by using collapsed molecules rather than individual read tags, which better reflects the ambient RNA generating process than previous attempts in the literature, which swapped individual read tags (13, 15). This strategy ensures that all the tags generated from a single molecule would be correctly swapped to a single cell and prevents the situation where some tags could remain in the original cell and some tags be distributed to one or more other cells.

### Transplant samples

To test scTx on “real-world” transplant samples, we generated scRNA-seq data from two chronic lung allograft dysfunction (CLAD) lung tissue samples at time of explant for re-transplantation and six post-lung transplant BAL specimens (18). Collection of samples was approved by the Research Ethics Board of University Health Network (#11-0509 and #15-9531). scRNA-seq data were generated using standard 10× Genomics scRNA-seq processes (see the Supplementary Material). Publicly available kidney transplant data from Malone et al. was downloaded as an additional dataset (7).

### Demultiplexing scRNA-seq samples using Vireo and Souporcell

We built a custom script as part of scTx to convert our SNV calls to the input required by Vireo and Souporcell. We applied minimal filtering to remove SNVs that are unlikely to be useful for demultiplexing: homozygous SNVs with an average minor allele fraction across all cells >0.999, and SNVs with low expression that have less than 10 cells covered or less than five cells with an alternative allele. SNV calls from the simulated and biological samples were annotated and filtered using the scSNV annotate tool. We then ran Vireo and Souporcell using two genotypes with the default parameters.

## Results

### Benchmarking scRNA-seq demultiplexing methods

To benchmark our scTx demultiplexing method against Vireo and Souporcell, we used our simulated post-transplant lung sample by mixing differing proportions of scRNA-seq PBMC and lung cells from independent and genetically distinct scRNA-seq samples (12 randomly generated mixtures of 4,000 singlet cells with 25–2,000 PBMCs) with simulated doublets equal to 10% of the minor genotype population and simulated ambient RNA contamination at a given rate (0%, 10%, 20%, 30%, and 40% ambient RNA contamination). Collectively, we simulated a total of 180 samples for benchmarking.

**Figure 2:**
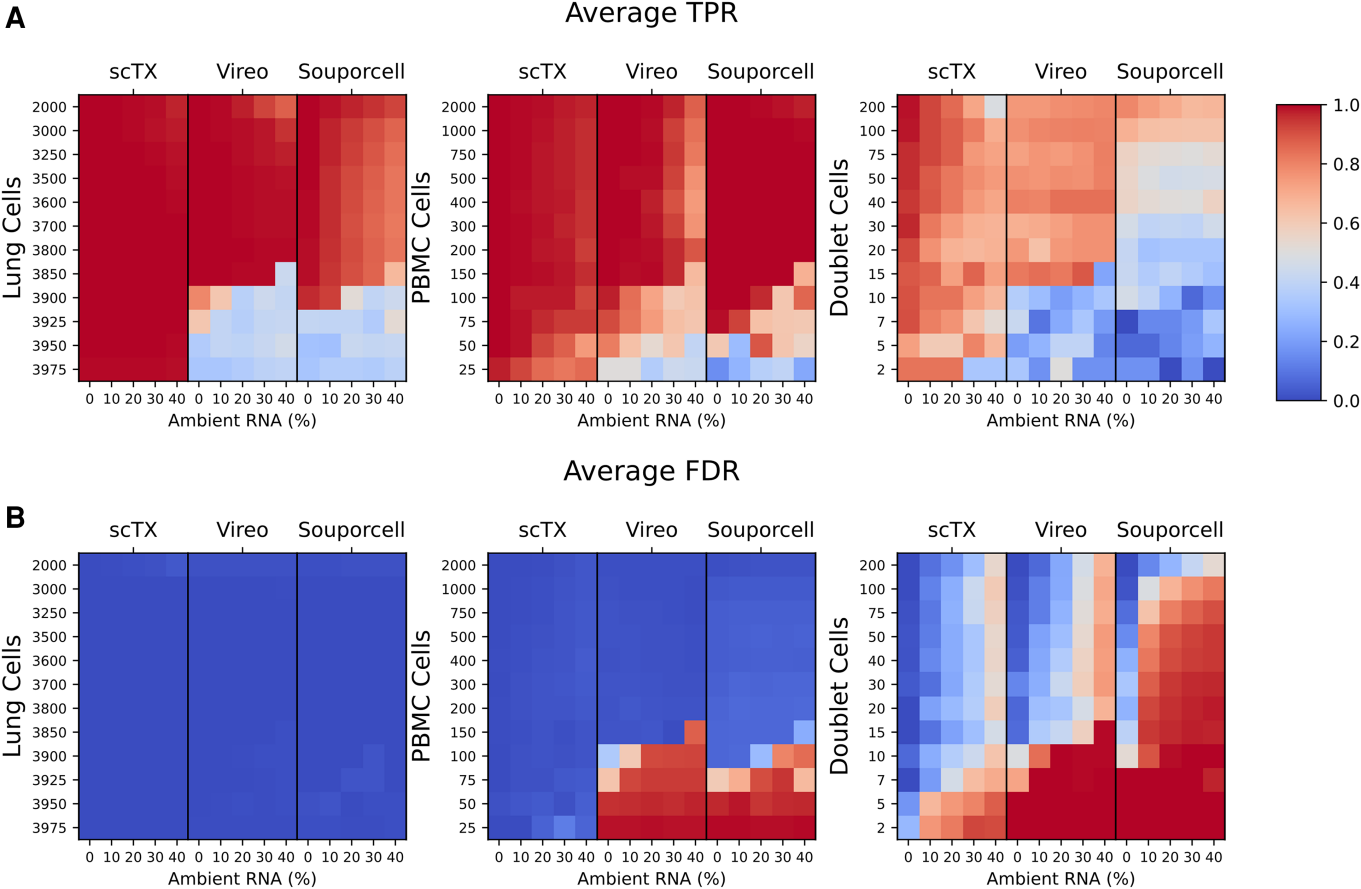
(**A,B**) Mean TPR and FDR values across all three replicates for the lung and PBMC mixture simulations. TPR and FDR values are grouped by their tool and ambient RNA(%) (columns). Each row represents a set of 12 mixtures from 25 to 2,000 PBMCs as indicated on the plots. The cell type used for calculating the TPR and FDR are indicated above each plot. TPR, true positive rate; FDR, false discovery rate.

We processed all 180 of the simulated SNV calls using scTx, Vireo, and Souporcell. There was a similar overall true positive rate (TPR) and false discovery rate (FDR) between all three methods across all replicates and ambient RNA contamination at PBMC counts greater than 100 (average TPR scTx: 0.996 ± 0.010, Vireo: 0.967 ± 0.088, and Souporcell: 0.923 ± 0.072; average FDR scTx: 0.008 ± 0.010, Vireo: 0.033 ± 0.088, and Souporcell: 0.077 ± 0.072). However, the performance of Vireo and Souporcell had a large drop in TPR and an increase in FDR when the number of PBMC cells were less than or equal to 100 (average TPR scTx: 0.996 ± 0.003, Vireo: 0.440 ± 0.159, and Souporcell: 0.456 ± 0.198; average FDR scTx: 0.004 ± 0.003, Vireo: 0.560 ± 0.159, and Souporcell: 0.544 ± 0.198) (Figure 2). Finally, we observed that all the tools had a lower overall doublet detection accuracy compared to their singlet detection accuracy and this is likely due to doublets generated from cell types with differing amounts of RNA; for example, a T cell/epithelial doublet would likely look more like the epithelial genotype due to the latter cell being larger and having more RNA content.

Overall, using our benchmarking pipeline, we observed that scTx was more robust to ambient RNA contamination and the number of PBMC cells when compared to Vireo and Souporcell, particularly when the number of PBMC cells was ≤100. We also observed that Vireo performed slightly better than Souporcell on our simulated data and that all the methods had reduced doublet detection efficiency compared to singlets.

### Donor and recipient demultiplexing on human kidney transplant samples

**Figure 3:**
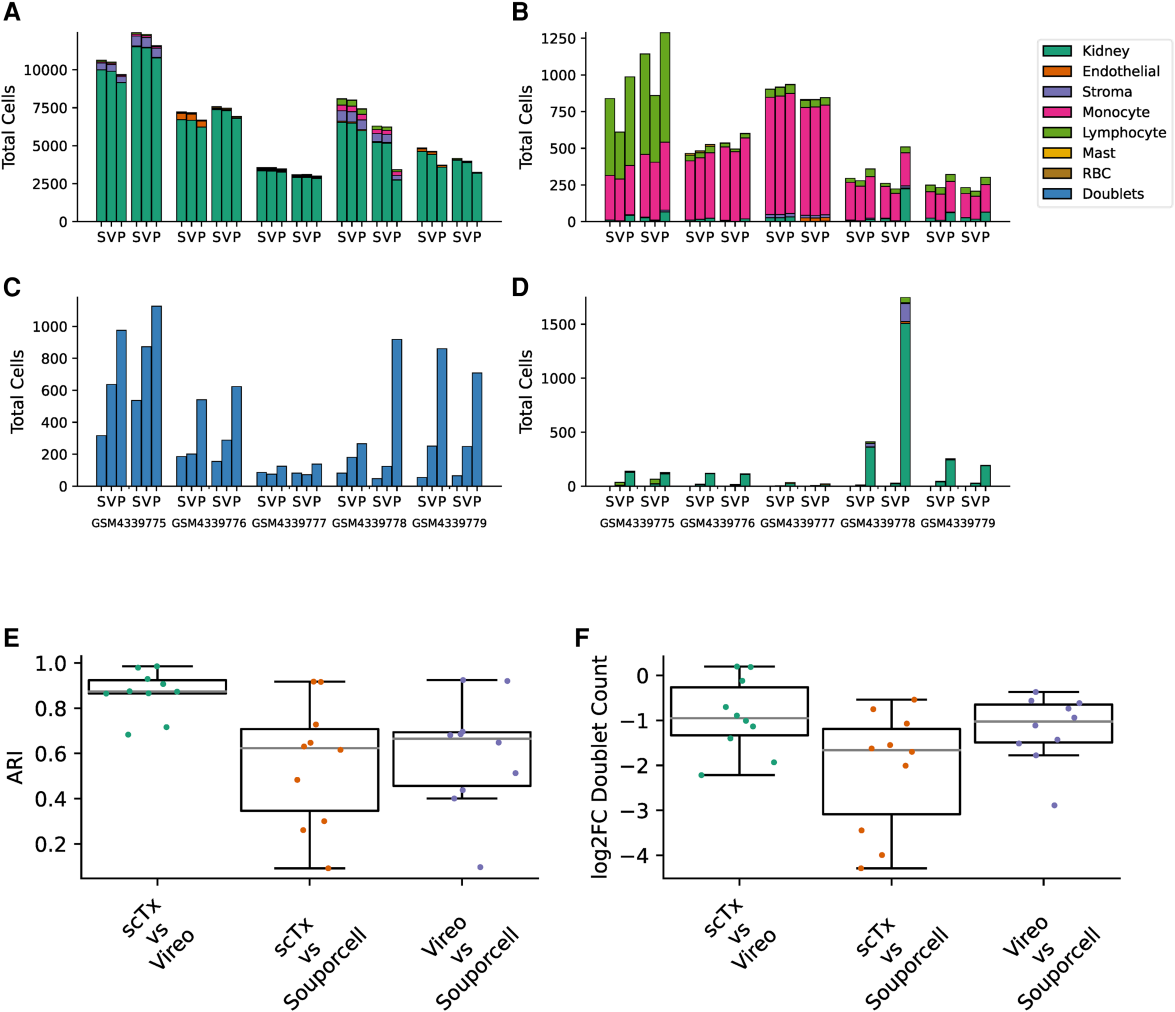
Summary of kidney sample demultiplexing using scTx (S), Vireo (V), and Souporcell (P). The total cells assigned to genotype 1 (**A**), genotype 2 (**B**), doublets (**C**), and unassigned (**D**), stratified by clustered cell type as per the legend. (**E**) The ARI was calculated for each sample between all the tool combinations as indicated on the *x*-axis. (**F**) The log_2_ fold change in the number of detected doublets between each tool combination as indicated on the *x*-axis. ARI, adjusted Rand index.

Having validated scTx using simulated data with a known makeup of cells, we next sought to utilize scTx on human transplant samples and compare its performance to Vireo and Souporcell. Publicly available scRNA-seq kidney transplant data from Malone et al. was downloaded and processed using scSNV and scTx with the same parameters as our simulation experiments (7, 15). For each of the five samples with two replicates each, cells were separated into “genotype 1” and “genotype 2” by each method and then clustered for cell type using community clustering. First, we examined the cell types associated with the genotype calls from each of the tools and observed that the one population was dominated by kidney parenchyma cells while the other was dominated with immune cells, which we expect from a reperfused graft (Figures 3A,B). Next, we looked at the similarity between the genotype assignments for each pair of tools using the adjusted Rand index (ARI), where a higher score indicates higher label assignment similarity. We observed that scTx and Vireo had a higher median score (0.874) versus comparisons with Souporcell (0.623 for scTx and 0.665 for Vireo) (Figure 3E). The major differences we observed were likely due to different doublet calling rates where scTx predicted the fewest doublets (median 1.9- and 3.16-fold fewer than Vireo and Souporcell, respectively) and Vireo observed an intermediary number (2.03-fold fewer than Souporcell) (Figure 3C and Figure 3F). We also observed that Souporcell failed on GSM4339778 replicate 2 where several hundred kidney cells were assigned to genotype 2 and a large number of cells were marked as unassigned (Figure 3D). This is consistent with our simulated data where we observed that Souporcell had the highest doublet FDR and lowest TPR. Given the reduced performance of Souporcell on both simulated and kidney datasets, we compared scTx only to Vireo for the remainder of the benchmarks.

### Visual confirmation of cell genotype assignments using dot plots

**Figure 4:**
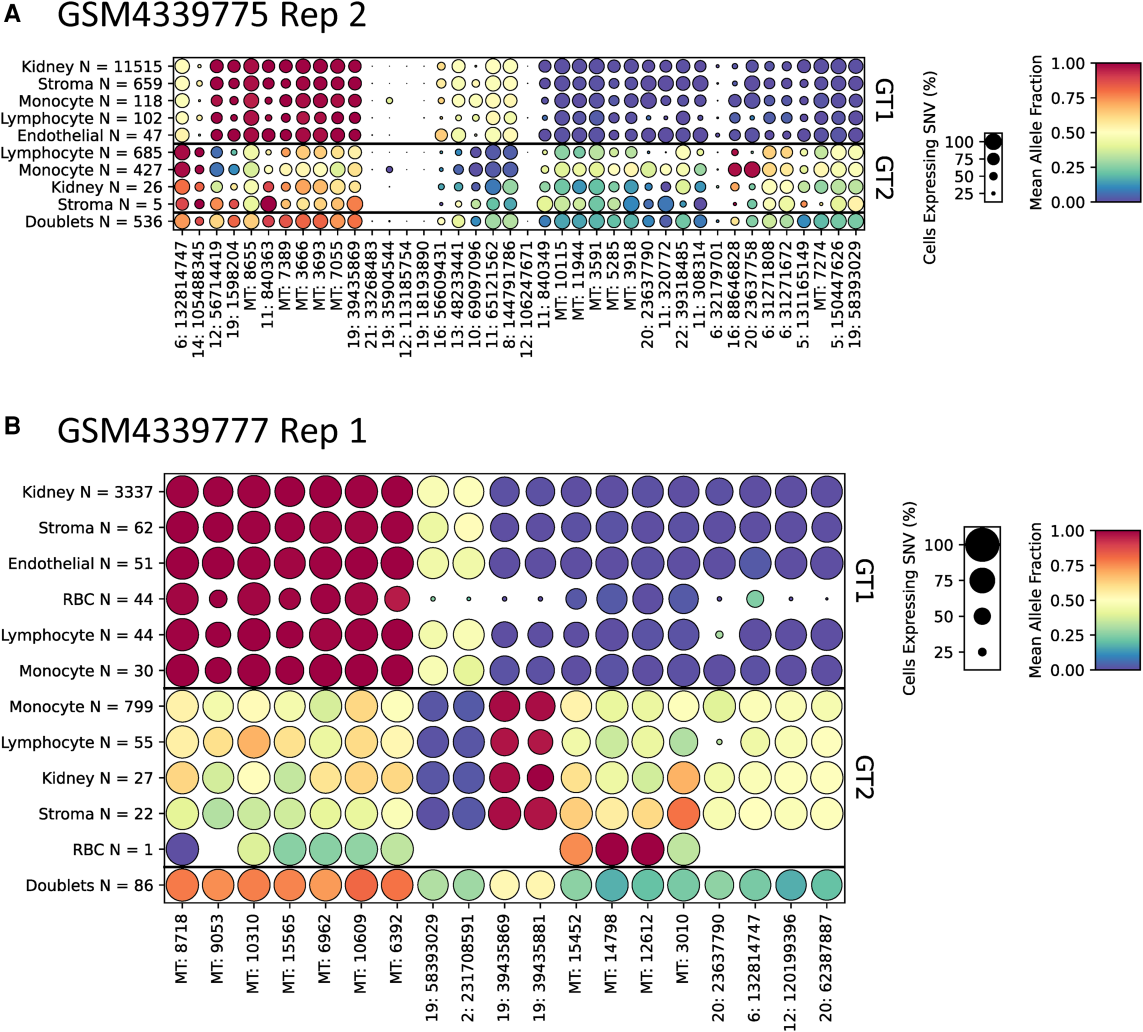
Representative dot plots from two kidney scRNA-seq samples GSM4339775 Replicate 2 (**A**) and GSM4339777 Replicate 1 (**B**). The size of each dot indicates the percentage of cells from that genotype/label class, the color of each dot shows the average allele fraction across all of the cells with expression at that locus. The genotype classes are indicated by the three row groups as labeled on the right side of the plots. The cell types and their counts are indicated for each row. Finally, the SNVs are represented by columns; the number prior to the colon represents the chromosome number or MT for mitochondrial. scRNA-seq, droplet-based single-cell RNA-sequencing

To aid in the interpretation of genotyping calls from scRNA-seq samples, we developed a method to find SNVs that are predictive of each genotype. This algorithm, included with scTx, can be used to find a set of SNVs that cover each cell at least N times (*N* = 5 by default) and are predictive of each genotype (see the Supplementary Material). To demonstrate this, we applied the tool to the kidney transplant samples using the scTx genotype calls (Figure 4 and Supplementary File S2). The results of these predictions can be visualized as dot plots where each column of dots is a set of predictive SNVs, the size of the dot represents the proportion of cells in each group and the color represents the mean allele fraction for each group. As expected, we observed large mean allele fraction differences between each genotype and the doublet calls tend to have mean allele fractions between the two individual genotypes. The most predictive SNVs for a given sample tend to be derived from mitochondrial reads, which is not surprising as these can represent a large proportion of a cell's sequenced molecules. This demonstrates that visualizing the genotyping results can be a powerful way to validate whether the tool has been successful at identifying the donor and recipient genotypes.

### Donor and recipient cell identification from lung transplant samples

**Figure 5:**
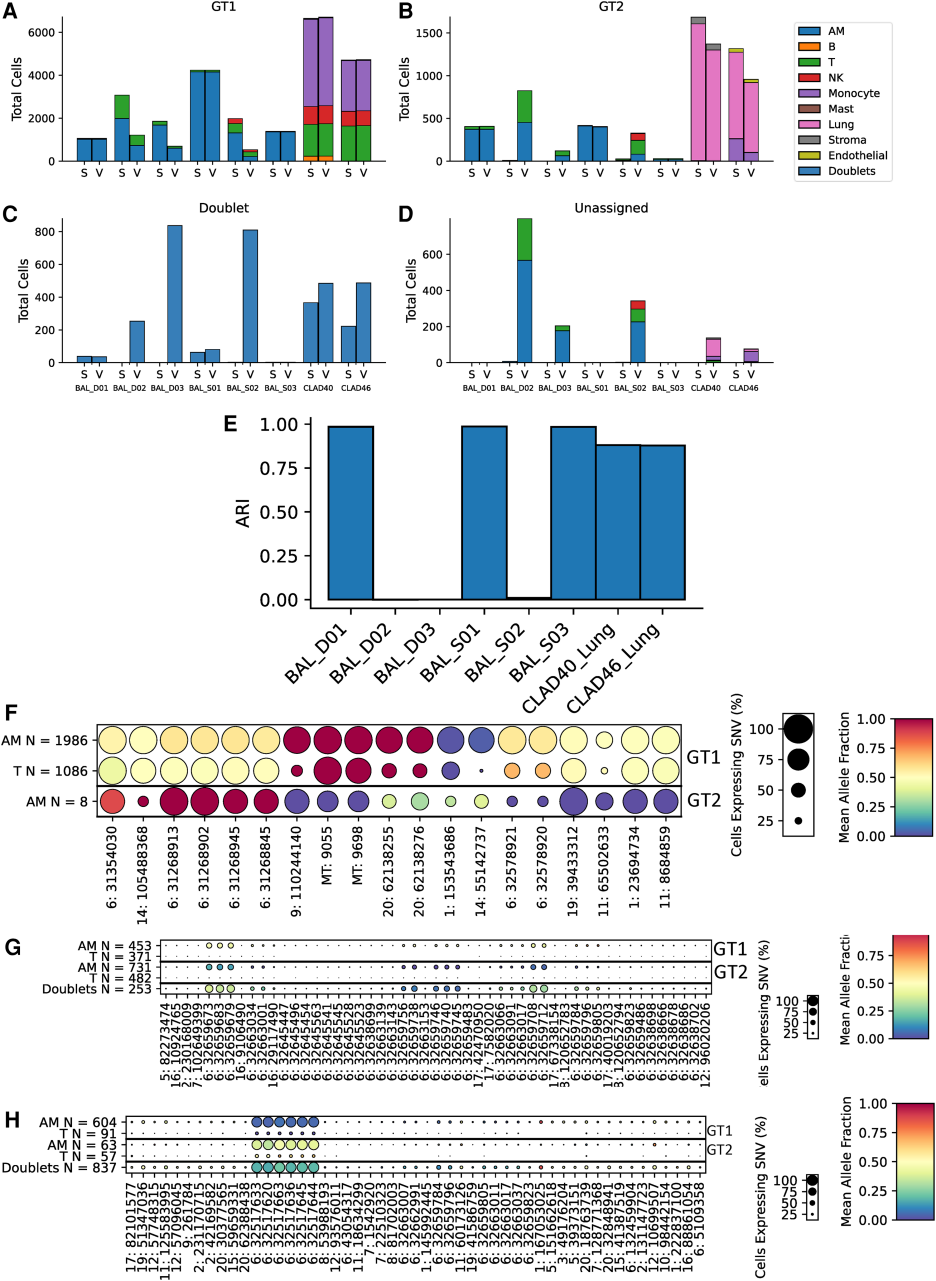
Cell and genotype assignment counts for GT1 singlets (**A**), GT2 singlets (**B**), doublets (**C**), and unassigned cells (**D**) using scTx (S) or Vireo (V). The six BAL samples and two CLAD samples are indicated on the *x*-axis, while the stacked bar charts show the number of cells from each cell type assigned to the given genotype as per the legend. Dot plots from BAL_D02 from scTx (**F**) and Vireo (**G)** and BAL_D03 with Vireo (**H**). The size of each dot indicates the percentage of cells from that genotype/label class, the color of each dot shows the average allele fraction across all of the cells with expression at that locus. The genotype classes are indicated by the three row groups as labeled on the right side of the plots. The cell types and their counts are indicated for each row. Finally, the SNVs are represented by columns. BAL, bronchoalveolar lavage; GT, genotype; CLAD, chronic lung allograft dysfunction; SNVs, single nucleotide variants.

To further assess scTx performance in transplant samples, we utilized eight samples from lung transplant recipients: six BAL and two lung allografts. The two lung allograft samples were similar to the kidney samples where the dominant (donor) genotype is parenchymal, and the minor (recipient) genotype expected to be circulating cells. However, the BAL samples provided a different challenge to the tools, where cell types are expected to be similar, but the minor genotype is usually rarer (Figures 5A–D). Surprisingly, we observed a large discrepancy between the scTx and Vireo calls on three of the six BAL samples where the adjusted Rand index was very low (Figure 5E). We suspected that these are cases where the number of cells in the minor genotype was low and Vireo generated artifactual genotype assignments. To verify this, we generated dot plots using our predictive SNV method for the genotype assignments from Vireo for BAL_D02 where scTx found eight cells (Figure 5F) from a second genotype and Vireo found 824 (Figure 5G). The sets of “predictive” SNVs found by the Vireo assignments clustered to a small region of chromosome six with low proportion of cells (small circles) and middling mean allele fractions (yellow color), suggesting that Vireo failed. In contrast, for the eight cells that scTx found, a SNV pattern consistent with the observation of a second genotype was seen. In this case, the eight cells from the second genotype provide information about how rare the minor genotype can be in these samples and how robust scTx is to rare genotypes. We did a similar analysis for BAL_D03 where scTx did not find a second genotype and Vireo found one with 120 cells. Again, we found a set of SNVs that were located within a similar region suggesting an artifactual genotype assignment by Vireo (Figure 5H). The remaining samples are shown in Supplementary File S3.

Collectively, we have highlighted that scTx can work on samples where the second genotype is very rare but also does not force a second genotype, if one cannot be found. It is important to validate the genotype assignments from a given tool using SNV data to verify that the assignment worked, and the dot plots from scTx facilitate this. Overall, scTx demonstrates superior performance for transplant sample genotype deconvolution when compared to existing methods.

## Discussion

A method for the robust segregation of donor from recipient cells is fundamental for the study of organ transplantation using scRNA-seq. Here, we demonstrate the utility of scTx as a current best practice method to differentiate donor and recipient cells in solid-organ transplant scRNA-seq samples using germline SNVs, without the need for costly germline sequencing of the donor or recipient. In order to adequately benchmark our algorithm, we required scRNA-seq data where the exact cell composition and cell type for each sample is known. As this cannot be generated experimentally, we created many sets of simulated transplant data *in silico*, combining PBMCs with lung parenchyma single-cell datasets to simulate a reperfused lung. This allowed us the flexibility to assess sensitivity by lowering the minor genotype down to 25 vs. 3,975 cells. We could also simulate common artifacts of droplet single-cell sequencing protocols, specifically doublets and ambient RNA, at varying percentages. Finally, this allowed us to objectively compare the performance of scTx—written specifically for transplant samples—to other current scRNA-seq deconvolution methods, Vireo and Souporcell.

Using the simulated data, we could show that scTx is accurate down to low numbers of the minor genotype cells, with a TPR of 1 down to 25:3,975 cells, whereas Vireo and Souporcell began to fail at 100:3,900 PBMC:lung cells. We could also demonstrate that scTx is accurate with increasing doublets even down to 5 doublets, whereas Vireo and Souporcell failed at around 10 and 50 doublets, respectively. As expected, ambient RNA also affected the ability of the algorithms to separate the two genotypes; however, Vireo and Souporcell demonstrated significantly hindered performance compared to scTx. This indicates that scTx will be more accurate in genotype segregation in real-world samples.

We next tested scTx on a number of scRNA-seq samples derived from human kidney transplants and lung allograft biopsies as well as BAL cells. We selected these samples as they represent common examples of transplant samples but also to highlight how transplant samples can differ in both cell type and composition. In the lung allografts, the donor parenchymal cells represent the major genotype and the circulating recipient cells are the minor genotype and the genotypes are largely different cell types. In BAL, the cell types between genotypes are similar, but the donor to recipient cell ratio is less predictable; this is similar to other liquid samples from solid-organ transplants such as urine or bile. We demonstrate that scTx is robust to these different samples and able to identify low numbers of the minor genotype when they exist (down to eight cells) and confidently call no other genotype when they may not exist. In contrast, Vireo missed the eight cells in that specific sample, and incorrectly identified a second genotype when one did not truly exist, in another.

An additional challenge with demultiplexing transplant cells using scRNA-seq data alone is the identification of which genotype represents the donor and which represents the recipient. For sample types where a large number of cells derive from the transplanted organ, the donor genotype can be inferred by cell type, i.e., lung origin cells are donor in a lung transplant. However, for some samples, such as our BAL samples, there were few parenchymal cells and the donor and recipient types must be inferred through other methods. For example, when the donor and recipient are sex-mismatched, the X- and Y-linked gene expression can be used. This needs to be considered during the design of the experiment.

The largest current limitation of scRNA-seq sample demultiplexing using SNVs is the detection of doublets, where all three methods had lower performance compared to singlet detection. Nonetheless, scTx still had better performance on our transplant samples compared to Vireo and Souporcell. The challenge with doublet detection is that they may appear to be more similar to singlets of one of the two genotypes depending on the two cells that were present in the droplet; for example, a large epithelial cell is likely to dominate in a doublet with a small T cell. This issue is further exacerbated by ambient RNA contamination which can bias a doublet even further to look like a singlet population, or cause a singlet cell to appear like a doublet. We demonstrated this by showing how ambient RNA affects our genotype scoring procedure in Figure 2. We recommend that users explore other doublet detection methods, such as Scrublet or DoubletFinder, that are based on gene expression and use these methods to further refine the doublet assignments generated by scTx (19, 20).

In this analysis, we utilized lung and kidney transplant samples, but scTx is generalizable to simulate and demultiplex samples from any transplant, or other samples where a mixture of two genotypes is expected. In conclusion, scTx is an objectively benchmarked and currently unparalleled best practice technique for the identification of donor and recipient cells from transplant scRNA-seq data. It can handle transplant datasets without need for genotyping separately either the donor and/or recipient and is robust in cases of differing cell types, which would be common in transplant samples. Moreover, it considers the confounding issues of doublets and ambient RNA contamination.

We anticipate that the availability of this mature bioinformatic process to analyze scRNA-seq data in the context of transplantation will spur the transplant community to embrace this technology for the study of donor–recipient interactions at a resolution previously unattainable.

## Data Availability

scTx is available at https://www.github.com/GWW/sctx.
